# Exercise activates Sirt1-mediated Drp1 acetylation and inhibits hepatocyte apoptosis to improve nonalcoholic fatty liver disease

**DOI:** 10.1186/s12944-023-01798-z

**Published:** 2023-03-07

**Authors:** Zongqiang Hu, Hongyu Zhang, Yiting Wang, Boyi Li, Kaiyu Liu, Jianghua Ran, Li Li

**Affiliations:** First People’s Hospital of Kunming City, The Calmette Affiliated Hospital of Kunming Medical University, Kunming, Yunnan China

**Keywords:** NAFLD, Aerobic exercise, Srit1, Drp1 acetylation, Mitochondrial dysfunction

## Abstract

**Purpose:**

Aerobic exercise has shown beneficial effects in the prevention and treatment of non-alcoholic fatty liver disease (NAFLD). Nevertheless, the regulatory mechanism is not turely clear. Therefore, we aim to clarify the possible mechanism by investigating the effects of aerobic exercise on NAFLD and its mitochondrial dysfunction.

**Methods:**

NAFLD rat model was established by feeding high fat diet. and used oleic acid (OA) to treat HepG2 cells. Changes in histopathology, lipid accumulation, apoptosis, body weight, and biochemical parameters were assessed. In addition, antioxidants, mitochondrial biogenesis and mitochondrial fusion and division were assessed.

**Results:**

The obtained in vivo results showed that aerobic exercise significantly improved lipid accumulation and mitochondrial dysfunction induced by HFD, activated the level of Sirtuins1 (Srit1), and weakened the acetylation and activity of dynamic-related protein 1 (Drp1). In vitro results showed that activation of Srit1 inhibited OA-induced apoptosis in HepG2 cells and alleviated OA-induced mitochondrial dysfunction by inhibiting Drp1 acetylation and reducing Drp1 expression.

**Conclusion:**

Aerobic exercise alleviates NAFLD and its mitochondrial dysfunction by activating Srit1 to regulate Drp1 acetylation. Our study clarifies the mechanism of aerobic exercise in alleviating NAFLD and its mitochondrial dysfunction and provides a new method for adjuvant treatment of NAFLD.

## Introduction

Chronic liver disease-non-alcoholic fatty liver (NAFLD) occurs worldwide, affecting an estimated 1.95 billion people worldwide [1, 2]. Liver injury, steatohepatitis, cirrhosis and fibrosis all fall under the umbrella of NAFLD and are associated with an increased risk of hepatocellular carcinoma and severe extrahepatic complications [3, 4]. Numerous studies have attested that the pathogenesis of NAFLD is intimately interrelated with mitochondrial dysfunction. Mitochondria play a role in regulating various cellular activities such as oxidative stress. Accumulation of intracellular lipids in NAFLD facilitates oxidative stress to generate superabundant reactive oxygen species (ROS), which gives mitochondrial function is affected and cytotoxic [5]. In contrast, mitochondrial dysfunction impairs fat homeostasis in the liver and results in the overproduction of ROS, which leads to a pernicious cycle that aggravates the evolution of NAFLD [6]. Recent studies have shown that regular physical exercise can improve NAFLD and its mitochondrial dysfunction [7], but its regulatory mechanism is not fully understood.

Sirtuin1 (Srit1) is a histone deacetylase involved in fatty acid synthesis, involved in fatty acid synthesis, oxidation, and adipogenesis [8]. Srit1 has been shown to play a beneficial role in mitigating NAFLD [9]. Activation of Srit1 significantly inhibits adipogenesis, attenuates high-fat diet (HFD)-induced inflammation and protects against hepatic steatosis in obese mice [10, 11]. Furthermore, Srit1 also has a momentous effect on protecting against mitochondrial dysfunction caused by NAFLD [12]. Activation of Srit1 can significantly reduce oxidative stress levels and ROS production in an in vitro-induced NAFLD model and alleviate mitochondrial dysfunction [13, 14]. The latest studies have indicated that exercise can increase the level of Srit1 [15, 16]. However, whether exercise alleviates NAFLD-induced mitochondrial dysfunction by elevating Srit1 expression remains unclear.

The balance of mitochondrial fission and fusion is essential for maintaining mitochondrial function [17]. Mitochondrial fission is normally regulated by dynamic-related protein 1 (Drp1), which functions by binding to receptors on the outer mitochondrial membrane, assembling into larger oligomers, and transporting to the fission site [18, 19]. Drp1 has been shown to promote mitochondrial fission through various posttranslational modifications, including phosphorylation, SUMOylation, and ubiquitination [20–22]. However, the acetylation of Drp1 remains unclear. Recent studies have shown that lipid overload leads to increased acetylation of DRP1 and enhances its activity, which in turn promotes mitochondrial fission and leads to cardiac dysfunction [23]. Srit1 is an inartificial histone deacetylase that exerts its deacetylation effect by removing the acetyl group that the latter adds to lysine residues to counteract the action of protein acetyltransferases [24]. Therefore, we postulate that aerobic exercise-activated Srit1 may alleviate NAFLD-induced mitochondrial dysfunction by regulating the level of Drp1 acetylation.

In this study, it probed the role of aerobic exercise in rats fed an HFD and the latent mechanisms. Our results showed that aerobic exercise alleviates liver injury and mitochondrial dysfunction induced by HFD in rats, and these effects are exerted by activation of Srit1 to inhibit Drp1 acetylation.

## Materials and methods

### Animal experiment

Three- to four-week-old male SD rats were purchased from the Animal Experimental Center of Kunming Medical University, kept in an immobile temperature and humidity environment, and adapted to a regular diet and drinking water for 7 days. There are normal control (NC) group and HFD group in this study, and the rats were randomly divided. In NC group, the rats were fed common rodent feed, and the proportion ratio of carbohydrate, fat, and protein was 7: 1: 2. The energy distribution proportion ratio of the HFD diet is 2: 6: 2. Rats in the NC group were fed continuously for 11 weeks until the end of the experiment. Obesity and metabolic syndrome characteristics began to develop in rats fed an HFD at the sixth week. After that, HFD rats were divided into three groups according to whether the animals received exercise training. The exercise program was based on a previous study [25]. One group of rats was divided into the HFD followed by aerobic exercise training (HFD + E) group, and the course consisted of a 10-min running warm-up, followed by resistance training, incorporating eight 2-min running sessions (with a 1-min rest interval) during which the rats ran at a slope, gradually increasing from 10 to 25° at an invariable slow speed (20–25 cm/s). Subsequently, persistent aerobic exercise was conducted for 30 min on the treadmill. The treadmill was purchased from Jiangsu SANS Biological Technology Co. Ltd. (product model: SA101). The second group of rats was injected with the Sirt1 inhibitor Tenovin-6 through the tail vein before aerobic exercise and then trained, which was called the HFD + E + T6 group. The third group of rats continued to be fed an HFD and remained sedentary. At week 11, the rats were sacrificed under anesthesia, and carotid blood and liver tissue were collected for subsequent analysis.

### Biochemical analysis of serum and liver tissues

Serum was prepared by centrifuging (4 °C, 5000 × g, 10 min) the collected rat blood and the serum was stored at -80 °C for subsequent analysis. We used PBS to flush the harvested liver tissue and using filter paper to wipe it. Then, we used formalin to immobilize the partial liver tissue at 4 °C, and a part of the liver tissue was freezed in liquid nitrogen and stored at -80 °C for subsequent analysis. Aspartate aminotransferase (AST), triglyceride (TG), and alanine aminotransferase (ALT) concentrations were detected by biochemical kits (Nanjing, China). Biochemical detection of SOD, GSH and MDA in liver tissue was also performed.

### Histopathological examination

For pathological testing, the right lobe of the liver was selected. The formalin-fixed liver tissue was sected, and before dyeing with hematoxylin and eosin (HE), it was cut into 6-μm slices and. Similarly, the liver stored at -80 °C were cut into 6 μm sections by a cryomicrotome. Then, oil red O solution and HE were used to dye the slices. Finally, light microscope was used to observe the histopathological structure.

### RNA extraction and analysis

TRIzol reagent (catalog number: 15596026, Invitrogen) was used to extract total RNA, and it was reversed to cDNA by a reverse transcription kit. Then, the SYBR Green Master Mix was used for RT‒qPCR. The reaction routine was as follows: pre-denaturation at 95℃ for 20 s; Then the amplification cycle was carried out at 95℃ for 1 s and 60℃ for 20 s, and there were 40 cycles in this stage. And then enter a dissolution curve analysis stage, wherein that temperature of the dissolution curve is set to be 60–95 deg C, and each sample is provided with three duplicate hole. Using β-actin as reference control, the level of the target product relative to the internal control was expressed as 2^−ΔΔCt^.

### Cell culture

In this study, the HepG2 cells (from the Cell Bank of the Chinese Academy of Sciences) were maintained in DMEM medium (containing 4.5 G/L glucose, 8.0% FBS, 100 U/mL penicillin and streptomycin). We used OA (Sigma‒Aldrich, catalog number: O1008) to treat cells. In brief, the parameters of the HepG2 cell incubator were: 37 °C and 5% CO_2_ for 24 h after seeding, whereas in the model, it was cultured for 48 h after treated with 1.2 mM OA to induce NAFLD. Cells were incubated with 20 μM CAY-10602 (Srit1 activator, MCE, catalog number: HY-104073) for 120 min whereas provoked with OA for 2 days. 12.0% serum was incorporated in the reconciled medium.

### CCK-8 detection of cell proliferation

In 96-well plates, HepG2 cells were treated with OA and CAY-10602 to a density of 5 × 10^3^ cells/mL in triplicate. In each well, 10 μL of CCK-8 was added after incubation for 2 days, and the cells were cultured for 60 min. The OD values detected under a microplate reader at 450 nm.

### HE and Oil Red O staining

For HE staining, we used 10% neutral formaldehyde to immobilize the cells for 20 min after removal of the cell culture medium. After flushed with PBS, the cells were dyed with HE for 1 min. After flushed by PBS, observing under a microscope and taking pictures. For Oil Red O staining, at 25 °C, the cells were dyed with Oil Red O solution (5 mg/mL) for 0.5 h. After flushed by PBS, observing under a microscope and taking pictures.

### Detection of apoptosis by flow cytometry

In this study, to check the apoptosis rate of HepG2 cells, we used the Annexin V-FITC/PI apoptosis kit. In brief, we used PBS to flush the cells, and the cells were digested and centrifuged with 1 × 10^4^ cells/mL. Using Annexin V-FITC and PI to hatch the cells. Finally, collecting the flow cytometry data and using FlowJo software to analyze it.

### Detection of intracellular ROS

In this study, we used an ROS detection kit to detect intracellular ROS production. Cells from each group were pretreated for 2 days, gathered and resuspended in 10 μM DCFH-DA solution (no serum). Then, at 37 °C, the samples were incubated for 20 min in darkness. After flushing with PBS, we used a fluorescence microscope to observe the intracellular fluorescence. Finally, to check the fluorescence intensity, which represents the ROS level, Image-Pro Plus 6.0 was used.

### Determination of adenosine triphosphate (ATP) content

We used an ATP bioluminescence assay kit to detect the cellular ATP levels according to the manufacturer's specifications.

### Mitochondrial membrane potential (MMP) measurement

To check the variation in MMP, we used an MMP assay kit with JC-1 in this study. At 37 °C, each group of cells was pretreated for 48 h, resuspended, and cultured with JC-1 for 20 min. Photographs were taken under a fluorescence microscope, and MMP changes were analyzed in light of the red/green fluorescence intensity ratio by Image-Pro Plus 6.0.

### RT‒qPCR of mitochondrial DNA (mtDNA) content

In this study, we used a QIaamp DNA mini kit to detect the total DNA, which was isolated from cells or frozen liver. Moreover, we used a Pico Green DNA quantification kit to detect the DNA concentration. Primers and FAM-labeled TAMRA quenching probes were purchased from TaKaRa Biotechnology. PCR detection kit was used for amplification and quantification of mtDNA.

### Immunofluorescence

In this study, we used prewarmed PBS to flush HepG2 cells 3 times and 4% paraformaldehyde to immobilize the cells for 20 min. Then, we used PBS (containing 5% BSA) to wash HepG2 cells for 60 min and used primary antibodies against nuclear respiratory factor 1 (NRF1; 1:200; ab200976; Abcam; UK) and Drp1 (1:250; ab184247; Abcam; UK) to incubate for 12 h at 4 °C. Subsequently, we used PBS to flush it 3 times and used secondary antibody goat anti-rabbit IgG (1:200, Abcam; ab150077; Abcam; UK) to hatch for 1 h at indoor temperature, protected from light. Nuclei were dyed with DAPI. Finally, we used Nikon Eclipse 80i microscope to observ sealed slides, and using Image-Pro Plus 6.0 to analyze the fluorescence intensity.

### Western blot

The liver tissue and HepG2 cells were lysed by RIPA lysis solution (Beyotime, China) to extract the protein. The protein concentration was detected by BCA reaction kit. After quantitative analysis, the total protein was denatured in this study. SDS-Page gel was used for electrophoresis, electrophoresis apparatus (Bio-RAD, USA) was adjusted to 120 V for electrophoresis, PVDF membrane (Millipore, USA) was used for membrane transfer, and skim milk (Sigma, USA) for blocking. Primary antibodies (Abcam, UK): Sirt1 (1:1000; ab189494), optic atrophy 1 (Opa1, 1:1000; ab157457), mitofusin 2 (Mfn2, 1:1000; ab124773), Drp1 (1:1000; ab184247), NRF1 (1:1000; ab34682), mitochondrial transcription factor A (TFAM, 1:1000; ab252432; Abcam; UK), Bcl-2 (1:2000; ab182858), cleaved-caspase 3 (1:500; ab2302), BCL2-associated X protein (Bax, 1:1000; ab32503), cleaved-caspase 9 (1:2000; ab32539) and GAPDH (1:2500; ab9485) were then added overnight to incubate. The next day, goat anti-rabbit antibody (1:2000; ab288151) was incubated for 1 h with slow shaking at 25 °C. The immunoreactive bands were visualized by intensive chemiluminescent reagent. The gray value was analyzed by ImageJ software.

### Statistical analysis

For all statistical analyses, statistical analyses GraphPad Prism7 was used. The analysis results are stand for the mean ± SD. One-way analysis of variance and t-test were used, and *P* < 0.05 were considered statistically significant.

## Results

### Aerobic exercise alleviates liver injury induced by HFD in rats

**Fig. 1:**
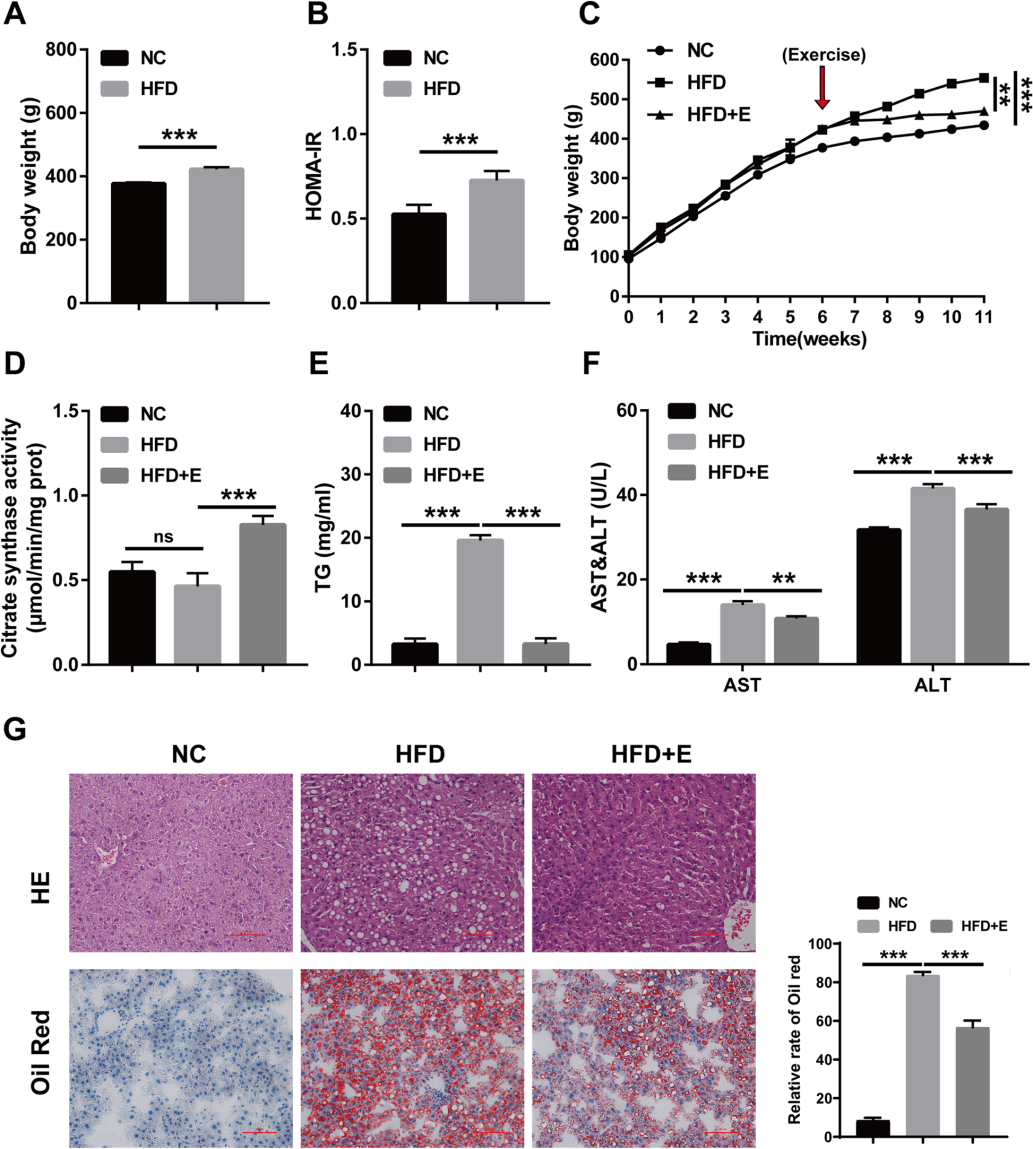
Effects of aerobic exercise on liver injury in HFD rats. **A** Body weight of rats after 6 weeks of HFD feeding; **B** Insulin resistance of rats after 6 weeks of HFD feeding; **C** Body weight monitoring of rats for 11 weeks; **D** Citrate synthase activity of rats after 11 weeks of HF feeding; **E** Blood lipid detection of rats after 11 weeks of HF feeding; **F** ALT and AST content of rat serum after 11 weeks of HF feeding; **G** HE and oil red O staining of rat liver tissue. (*P < 0.05, **P < 0.01, *** *P* < 0.001; ns: no significant difference)

NAFLD model was established in rats fed with HFD to determine the influence of aerobic exercise on NAFLD-induced liver injury. SD rats aged 3–4 weeks were fed a normal or HFD diet, and after 6 weeks, the rats began to develop early obesity and metabolic syndrome, such as increased body weight and insulin resistance (Fig. 1A-B). Then, the experiment was performed in the following groups: the NC group (normal control diet), HFD group (fed HFD and sedentary), and HFD + E group (fed HFD followed by forced aerobic exercise). Rat body weights were measured weekly during the 11 weeks of feeding. After the last training session, rats were executed under anesthesia for subsequent analysis. The results of continuous weight monitoring for 11 weeks showed that HFD caused significant weight gain in rats compared with the NC group, and aerobic exercise could alleviate the weight gain caused by HFD (Fig. 1C). Citrate synthase activity was evaluated as a certification of notion to validate the effectiveness of the exercise. Therefore, we examined the activity of citrate synthase in rat serum, and the obtained results showed that citrate synthase activity was not arrested in the NC and HFD groups, while citrate synthase activity was prominently enhanced in the HFD + E group (Fig. 1D). Another biochemical test showed that HFD caused a significant increase in blood lipids in rats, and aerobic exercise could prevent HFD-induced dyslipidemia (Fig. 1E). The results of serum ALT and AST tests showed that long-term feeding of HFD led to a prominent enhancement in the content of ALT and AST in rats, while aerobic exercise could reduce the content of ALT and AST in serum (Fig. 1F). In addition, HE staining of rat liver tissue showed that rats fed an HFD for 11 weeks showed a typical appearance of fatty acid infiltration in the liver, indicating that fat metabolism was disrupted, and aerobic exercise effectively alleviated these pathological characteristics. The lipid droplets in HFD + E group were smaller and lower in content, indicating that lipid deposition in liver was alleviated (Fig. 1G).

### Aerobic exercise improves HFD-induced decreased Sirt1 expression and mitochondrial dysfunction in rats

**Fig. 2:**
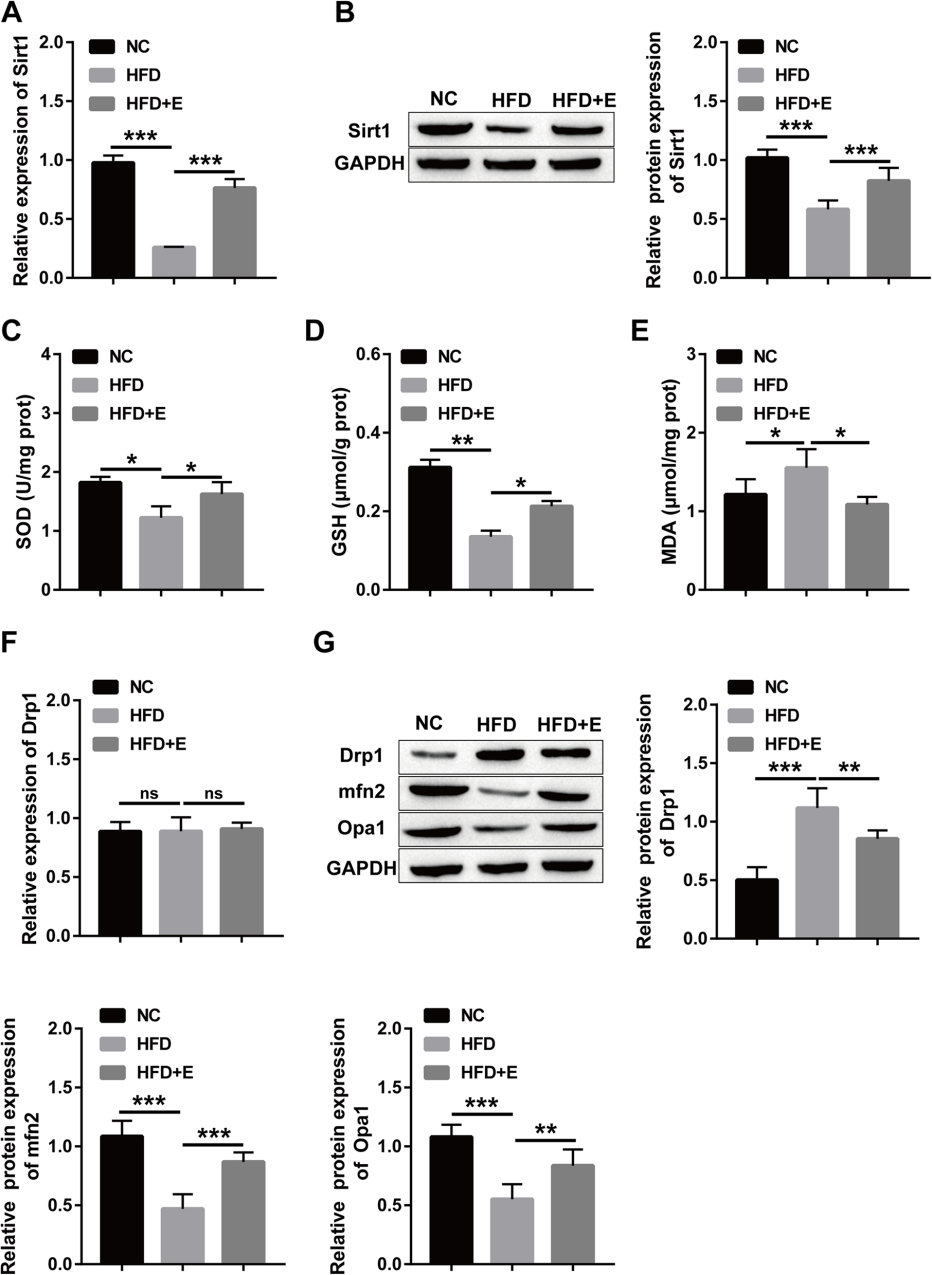
Aerobic exercise ameliorates HFD-induced reduction of Sirt1 expression and mitochondrial dysfunction in rats. **A** RT‒qPCR was used to detect the mRNA level of Sirt1; **B** Western blot for the protein level of Sirt1; **C**-**E** Kit for the contents of SOD, GSH and MDA; **F** RT‒qPCR for the mRNA level of Drp1; **G** Western blot for the protein level of Drp1, Opa1 and Mfn2. (*P < 0.05, **P < 0.01, *** *P* < 0.001; ns: no significant difference)

It has been confirmed that aerobic exercise can alleviate liver injury in rats. Sirt1 levels in liver tissue were detected to explore the mechanism of aerobic exercise to improve liver injury in rats. Obviously, the level of Sirt1 in rat liver tissue decreased after high fat induction, and the expression of Sirt1 was restored after aerobic exercise (Fig. 2A-B). Subsequently, the results of oxidative stress level and antioxidant enzyme activity in rat liver tissue showed that HFD caused a remarkable decrease in SOD and GSH and a prominent increase in MDA, and these were reversed by aerobic exercise (Fig. 2C-E). Mitochondrial fission and fusion are indispensable for the maintenance of form and function. Hence, RT‒qPCR was used to check the influence of aerobic exercise on mitochondrial dysfunction induced by HFD. However, we did not observe significant changes in the gene (Fig. 2F). But, at the protein level, it was found that Drp1 expression increased and Mfn2 and Opa1 expression decreased. Aerobic exercise could reverse the protein changes (Fig. 2G).

### Aerobic exercise reverses the HFD-induced increase in Drp1 acetylation in rats

**Fig. 3:**
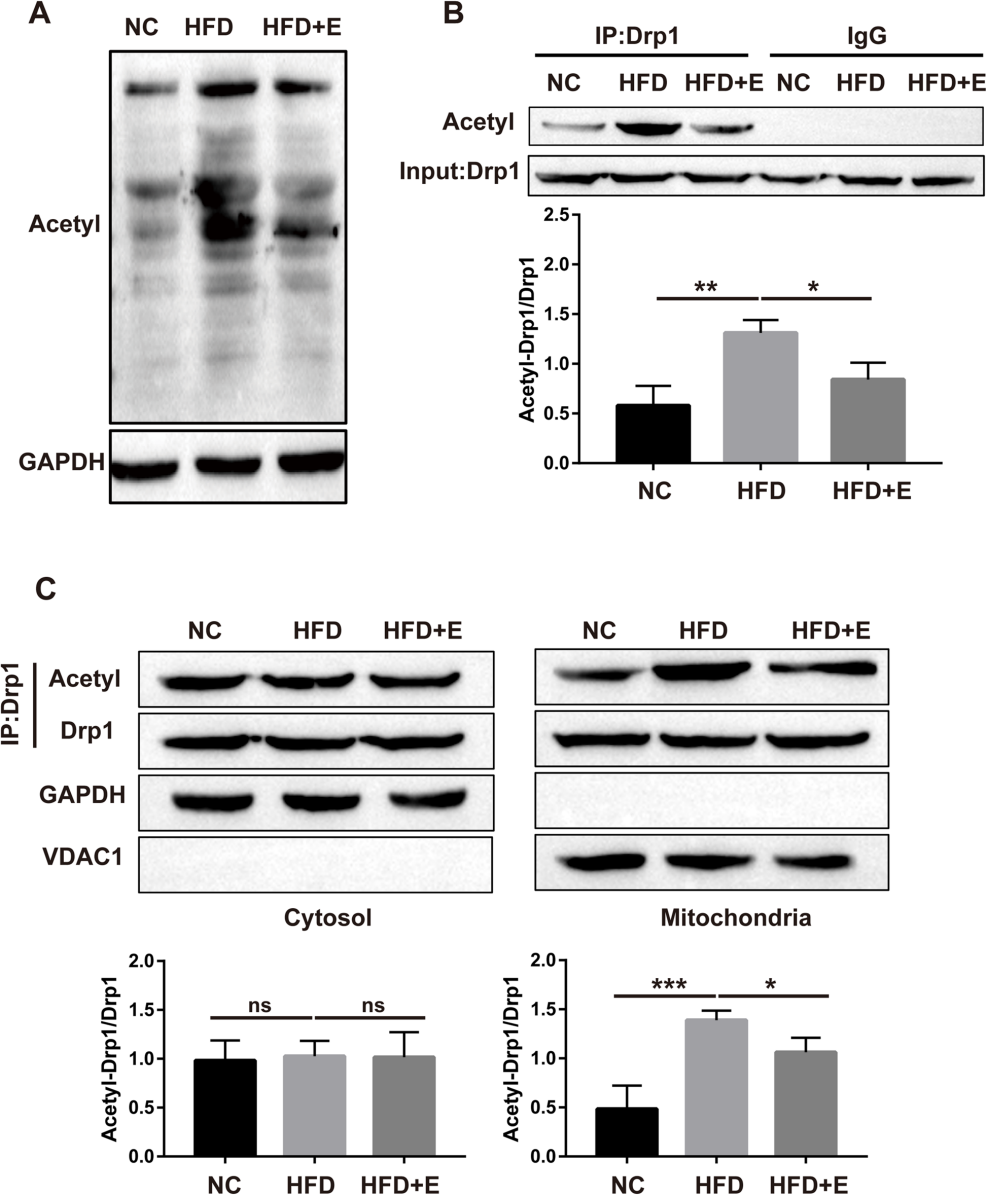
Aerobic exercise reverses the HFD-induced increase in Drp1 acetylation in rats. **A** Western blot was used to detect the acetylation level of total protein in rat liver; **B** Western blot for the acetylation level of Drp1 in rat liver; **C** Western blot for the acetylation levels of Drp1 in cytoplasm and mitochondria. (*P < 0.05, **P < 0.01, *** *P* < 0.001; ns: no significant difference)

Next, we investigated the mechanism by which aerobic exercise downregulates Drp1. Since HFD feeding did not alter the mRNA levels of Drp1 in the liver, it is suggested that transcriptional regulation is not necessarily the latent mechanism. Therefore, we speculated that the posttranslational embellishment of Drp1 may be the cause of the elevated level of Drp1 protein. To test this assumption, we checked the acetylation levels of liver total protein and Drp1 in rats fed an HFD, and high fat induction increased the total protein content and the acetylation level of Drp1 in the liver, and these conditions were also reversed by aerobic exercise (Fig. 3A-B). Subsequently, we examined the acetylation of Drp1 in the cytoplasm and mitochondria. The HFD induced elevated Drp1 acetylation chiefly in the mitochondrial fraction rather than the cytosol, suggesting that acetylation was interrelated with Drp1 translocation to the mitochondria, whereas aerobic exercise decreased the level of Drp1 acetylation in the mitochondria (Fig. 3C).

### Mitochondrial dysfunction was alleviated by aerobic exercise

**Fig. 4:**
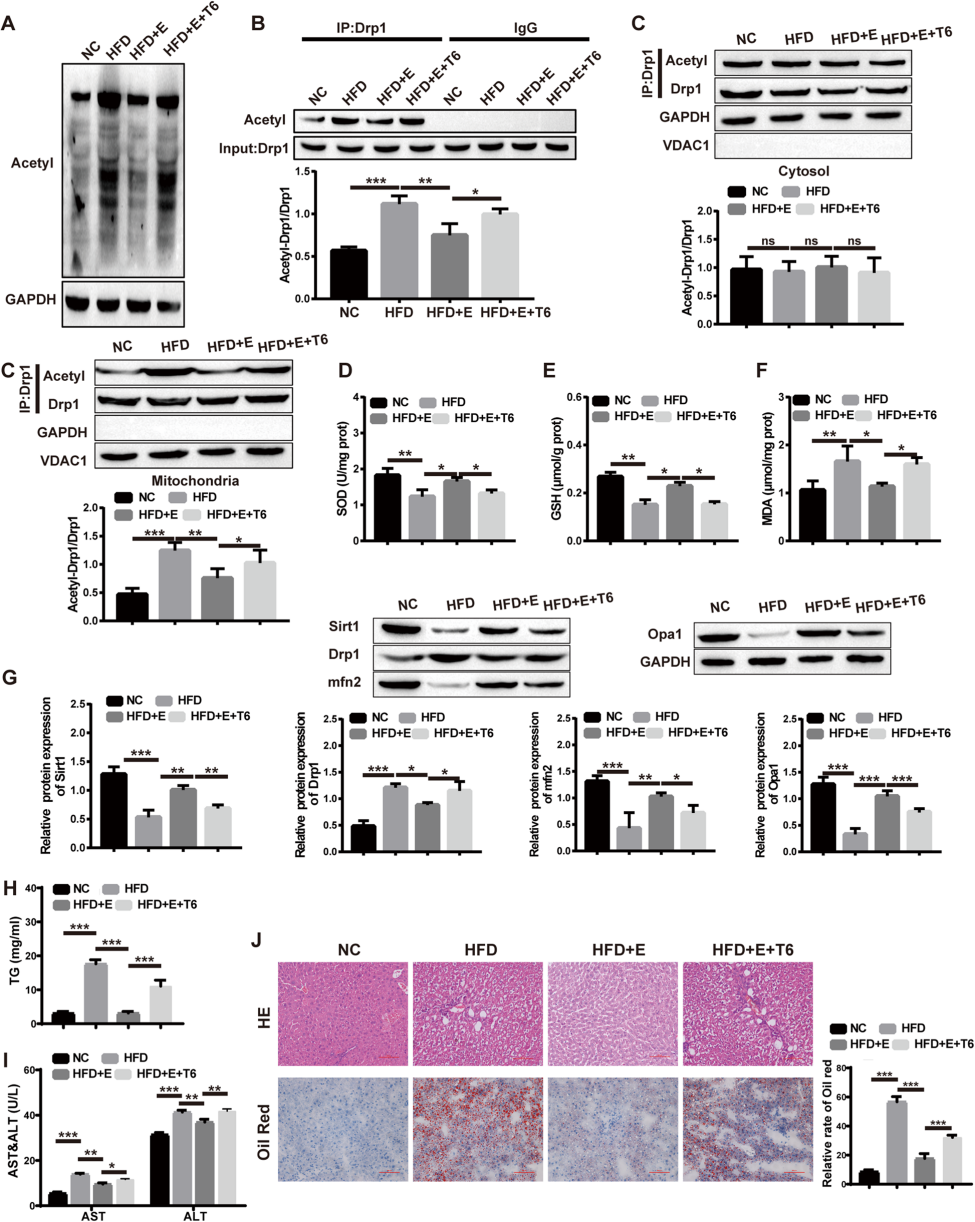
Sirt1 inhibitors reverse aerobic exercise-reduced Drp1 acetylation and mitochondrial dysfunction. **A** Western blot for detecting the acetylation level of total protein; **B** Western blot for the acetylation level of Drp1; **C** Western blot for the acetylation levels of Drp1 in cytoplasm and mitochondria; D-F: Kit for the contents of SOD, GSH and MDA; **G** Western blot for the protein level of Sirt1, Mfn2, Drp1 and Opa1; **H** Blood lipid detection of rat; **I** ALT and AST content of rat serum; **J** HE and oil red O dying of rat liver tissue. (T6, tenovin-6). (*P < 0.05, **P < 0.01, *** *P* < 0.001; ns: no significant difference)

Since Sirt1 is a natural histone deacetylase, aerobic exercise activates the expression of Sirt1. As a consequence, we deduced that aerobic exercise may downregulate the level of Drp1 by activating Sirt1 to reduce Drp1 acetylation. To test this hypothesis, we injected the Sirt1 inhibitor Tenovin-6 into the tail vein of rats before aerobic exercise training to observe its effects on Drp1 acetylation and mitochondrial dysfunction. The acetylation results of liver total protein and Drp1 showed that tenovin-6 reversed the acetylation reduction of total protein and Drp1 by aerobic exercise (Fig. 4A-B). Similarly, inhibitors reverse the high lipid-induced mitochondrial Drp1 acetylation levels (Fig. 4C). In addition, Tenovin-6 also reversed the increase in SOD and GSH levels, as well as the decrease in MDA levels, caused by aerobic exercise (Fig. 4D-F). Next, we found that the expression of Sirt1, Mfn2 and Opa1 proteins decreased and Drp1 protein increased after treatment with the inhibitor on the basis of aerobic exercise (Fig. 4G). In addition, high fat diet can increase the content of TC, ALT and AST in rats, while aerobic exercise could reduce the contents of TC, ALT and AST in serum, and Sirt1 inhibitor reversed the abovementioned results (Fig. 4 H-I). In addition, the HE staining results of rat liver tissuewas infiltrated by fatty acids in the liver, and aerobic exercise effectively alleviated these symptoms. The Sirt1 inhibitor reversed this effect. compared with the HFD group, the lipid droplets in the HFD + E group decreased in size and number, indicating that liver lipid deposition was relieved, and the addition of the Sirt1 inhibitor aggravated liver lipid deposition (Fig. 4J). In short, our results showed that Sirt1 inhibitors reversed the reduced Drp1 acetylation and mitochondrial dysfunction associated with aerobic exercise.

### Sirt1 inhibits OA-induced damage in HepG2 cells

**Fig. 5:**
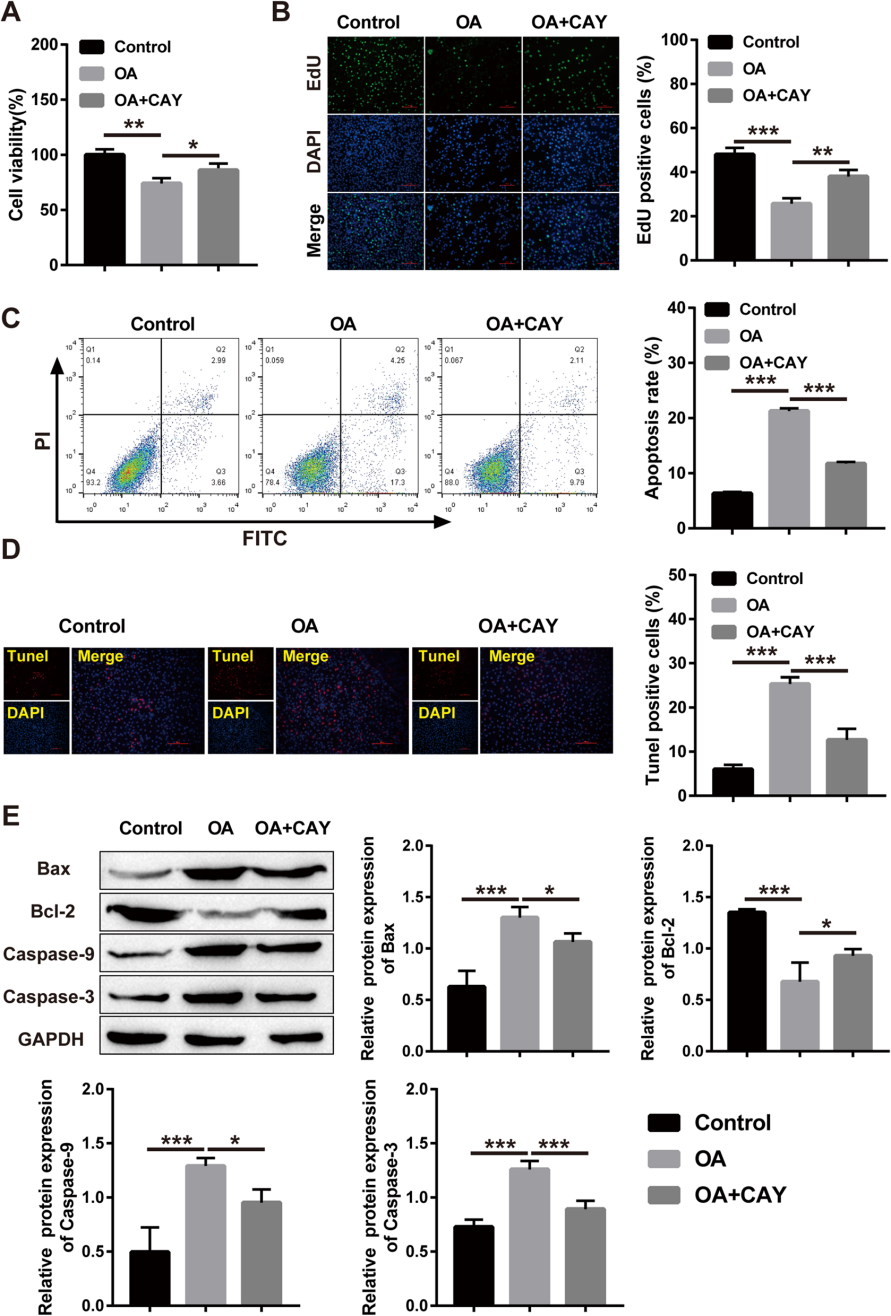
Sirt1 inhibits OA-induced damage in HepG2 cells. **A** CCK-8 for checking HepG2 cell viability; **B** EdU for HepG2 cell proliferation; **C** Flow cytometry for HepG2 apoptosis; **D** TUNEL staining for HepG2 apoptosis; **E** Western blot for the level of proteins Bax, caspase-9, caspase-3 and Bcl-2. (*P < 0.05, **P < 0.01, *** *P* < 0.001; ns: no significant difference)

Cell activity and toxicity were detected by CCK8 assay. The obtained results showed that the cell vigor was memorably lessened after OA treatment, while the addition of a Sirt1 activator (CAY-10602, CAY) rescued the low cell vigor caused by OA (Fig. 5A). The results of EdU detection showed that the cell proliferation was markedly lessened after OA remedy, and cell proliferation capacity was observably elevated after adding the Sirt1 activator (Fig. 5B). Subsequently, we validated the influence of Sirt1 activators on apoptosis in HepG2 cells. Flow cytometry results showed that apoptosis was elevated after OA treatment, and the Sirt1 activator rescued OA-induced apoptosis (Fig. 5C). TUNEL and WB showed similar results, with OA treatment increasing apoptosis and the Sirt1 activator reversing this trend (Fig. 5D-E). Taken together, our results indicate that the Sirt1 activator protects against OA-induced injury in HepG2 cells.

### Sirt1 can affect the process of lipid accumulation and mitochondrial damage induced by OA

**Fig. 6:**
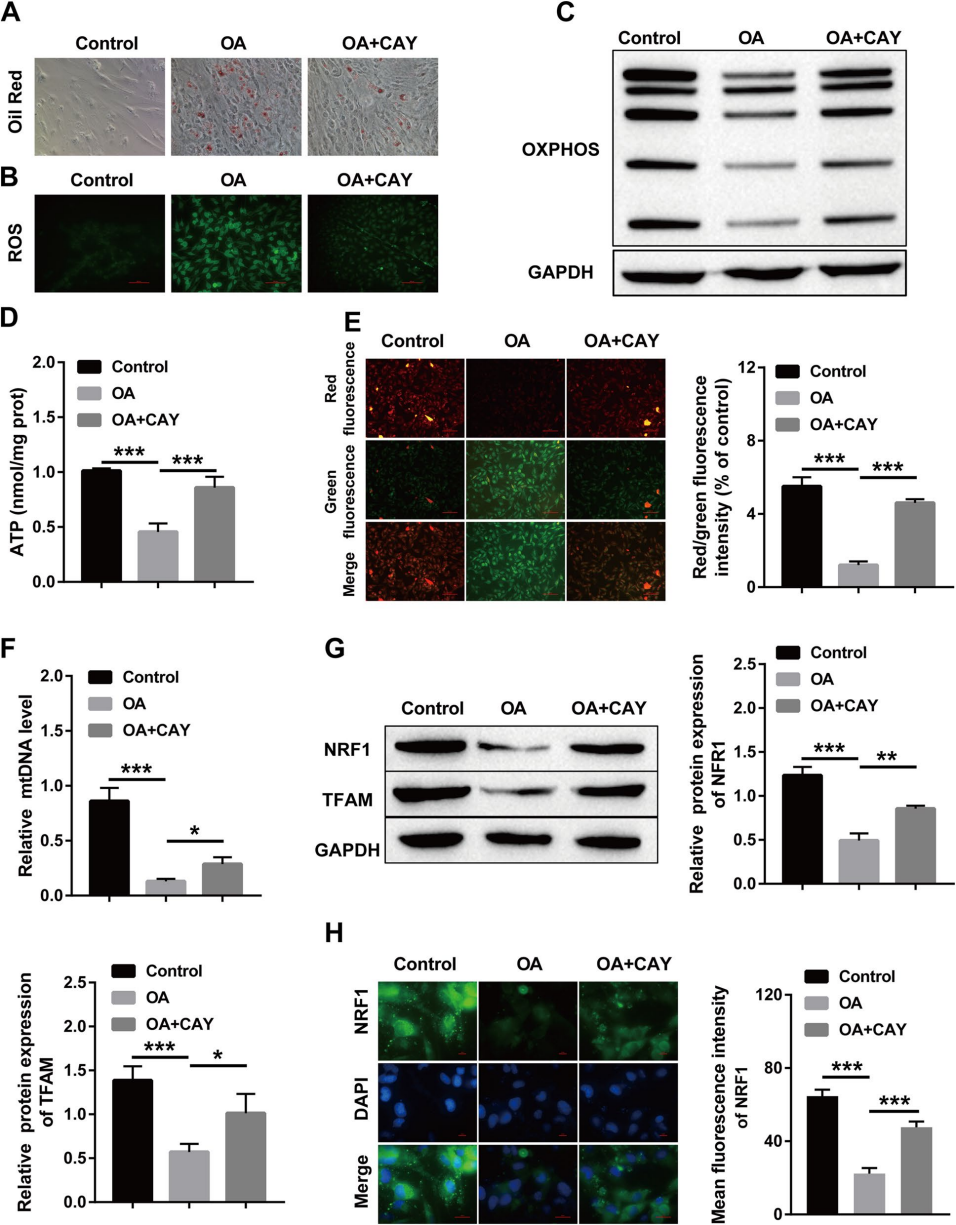
Sirt1 attenuates OA-induced lipid accumulation and mitochondrial dysfunction in HepG2 cells. **A** Oil red O staining for observing intracellular lipid accumulation; **B** immunofluorescence staining for checking intracellular ROS level; **C** Western blot for intracellular OXPHOS expression; **D** ATP production in cells was detected by kit; **E** mitochondrial membrane potential (ΔΨm) changes were detected by kit; **F** The copy number of mtDNA related to mitochondrial genesis was detected by kit; **G** Western blot for the level of NRF1 and TFAM; H: Immunofluorescence staining for the level of NRF1. (*P < 0.05, **P < 0.01, *** *P* < 0.001; ns: no significant difference)

HepG2 cells were treated with OA to construct NAFLD model in vitro, and the relationship between Sirt1 and lipid accumulation and mitochondrial function was further verified. The results showed that OA treatment led to lipid accumulation in HepG2 cells, which was manifested as intracellular red oil droplets, and the addition of a Sirt1 activator attenuated OA-induced lipid accumulation (Fig. 6A). To assess mitochondrial injury, intracellular ROS levels were visualized by immunofluorescence staining. The obtained results showed that the intracellular ROS level was observably elevated after OA treatment, and the addition of a Sirt1 activator decreased OA-induced ROS production (Fig. 6B). Oxidative phosphorylation (OXPHOS) is a momentous index of mitochondrial function. The Western blot results of OXPHOS showed that the OXPHOS complex was reduced after OA treatment, while OXPHOS enzymatic activity was restored after the addition of the Sirt1 activator (Fig. 6C). Mitochondria are known to be major sites of ATP production, and we checked energy production in OA-treated and decreased ATP production, which was restored by the addition of the Sirt1 activator (Fig. 6D). Similarly, OA treatment decreased the mitochondrial membrane potential (ΔΨm), whereas the addition of a Sirt1 activator restored ΔΨm (Fig. 6E). In addition, OA treatment also decreased the mitochondrial biogenesis-related mtDNA copy number, which was restored by the addition of the Sirt1 activator (Fig. 6F). We also found that OA treatment decreased the protein levels of NRF1 and TFAM in HepG2 cells, and these results were reversed by the addition of a Sirt1 activator (Fig. 6G). Similarly, immunofluorescence showed the same trend, with OA treatment leading to a decrease in NRF1 fluorescence intensity and the addition of a Sirt1 activator leading to an increase in NRF1 expression (Fig. 6H).

### OA-induced increase in Drp1 expression in HepG2 cells is caused by increased acetylation levels

**Fig. 7:**
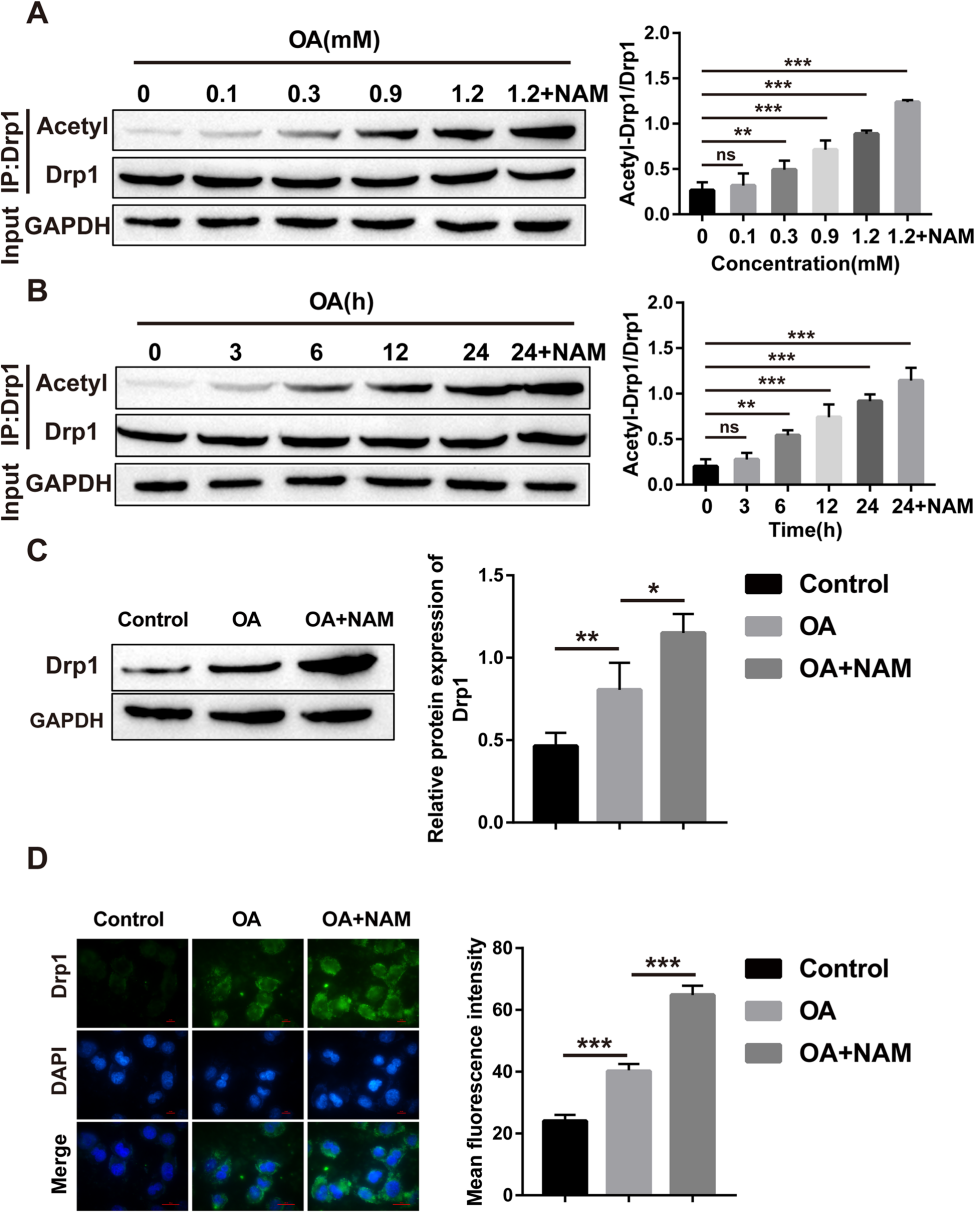
OA-induced increased expression of Drp1 in HepG2 cells is caused by increased acetylation levels. **A** The effect of OA concentration and NAM on the acetylation level of Drp1; **B** The effect of OA treatment time and NAM on the acetylation level of Drp1; **C** Western blot for the protein level of Drp1; **D** Immunofluorescence staining for the level of Drp1. (*P < 0.05, **P < 0.01, *** *P* < 0.001; ns: no significant difference)

Next, we examined the level of Drp1 acetylation in OA-treated HepG2 cells. The obtained results showed that the acetylation level of Drp1 increased with increasing OA remedy concentration and treatment time and further increased after the addition of nicotinamide (NAM, deacetylase inhibitor) (Fig. 7A-B). In addition, Drp1 protein levels were observably elevated after OA treatment and further increased after the addition of NAM (Fig. 7C-D). In summary, our results suggest that the OA-induced elevation of Drp1 expression is caused by increased acetylation levels.

### Sirt1 regulates Drp1 acetylation and affects lipid accumulation and mitochondrial dysfunction

**Fig. 8:**
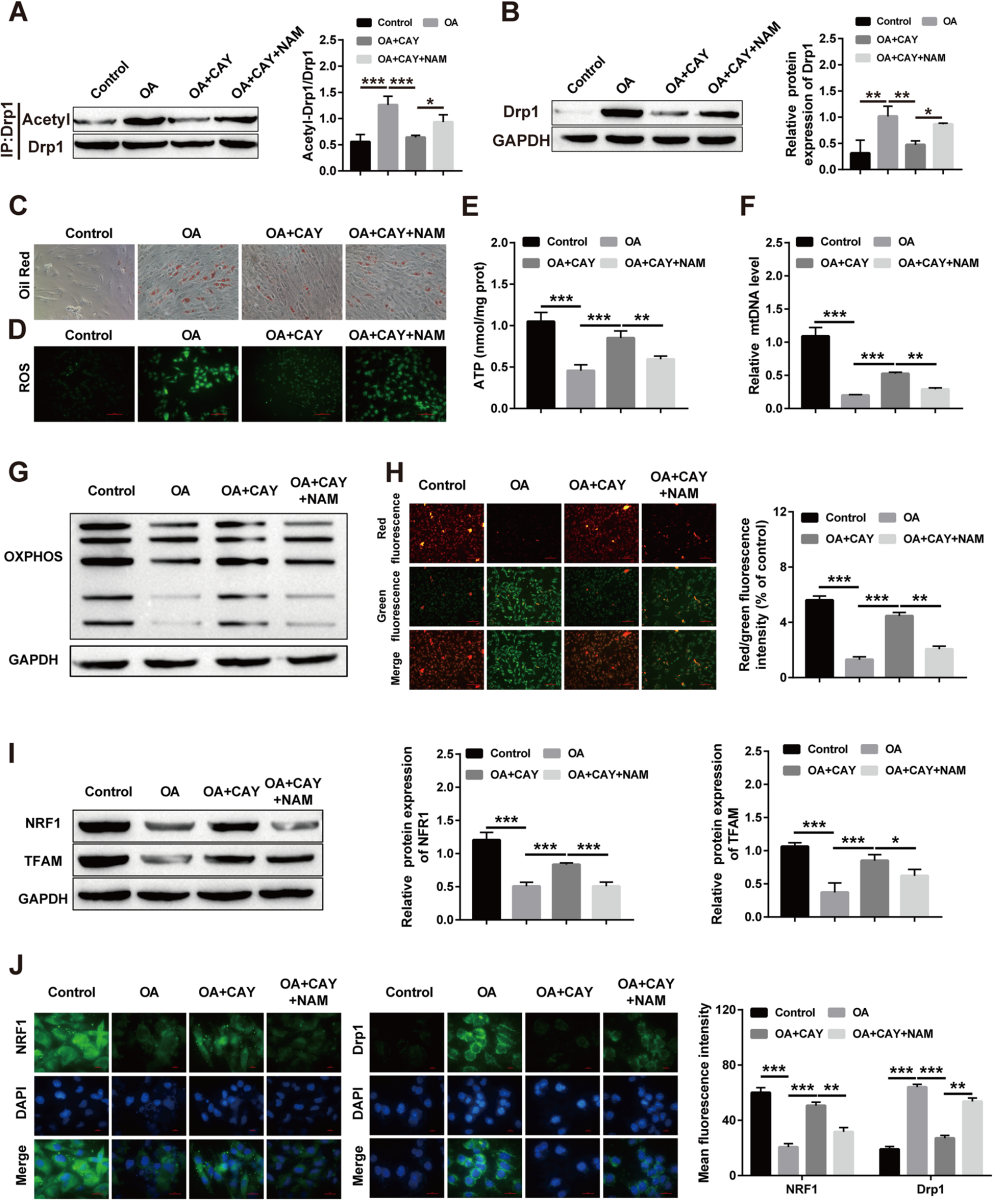
Sirt1 attenuates OA-induced lipid accumulation and mitochondrial dysfunction in HepG2 cells by regulating Drp1 acetylation. **A** Western blot for Drp1 acetylation; **B** Western blot for Drp1 protein expression; **C** Oil red O staining for lipid accumulation; **D** Immunofluorescence for ROS content; **E**–**H** Kit for ATP, mtDNA, OXPHOS and ΔΨm levels; **I** Western blot for NRF1 and TFAM protein levels; immunofluorescence for NRF1 and TFAM levels. (*P < 0.05, **P < 0.01, *** *P* < 0.001; ns: no significant difference)

Finally, we added NAM simultaneously with a Sirt1 activator to OA-induced HepG2 cells to test the influence of Drp1 acetylation. Western blot results showed that the Sirt1 activator-induced reduction in Drp1 acetylation was reversed by the addition of NAM (Fig. 8A). The obtained results also showed that the Sirt1 activator-induced decrease in Drp1 protein levels were reversed by the addition of NAM (Fig. 8B). Oil red O staining showed that the addition of NAM inhibited the Sirt1 activator-induced reduction in lipid accumulation (Fig. 8C). Intracellular ROS assays showed that the addition of NAM restored the intracellular ROS decreased by the Sirt1 activator (Fig. 8D). In addition, ATP, mtDNA, OXPHOS, and ΔΨm assays showed that the salutary influence of Sirt1 activators on mitochondrial biogenesis, mitochondrial fusion and division was rescued by the addition of NAM (Fig. 8E-H). The obtained results indicated that the increased NRF1 and TFAM protein expression induced by Sirt1 activator treatment was suppressed by the addition of NAM (Fig. 8I). Immunofluorescence also showed similar results, with NAM treatment reversing the increase in NRF1 fluorescence intensity, and the decrease in Drp1 fluorescence intensity is caused by Sirt1 activators (Fig. 8J). In short, our results suggest that NAM supplementation can regulate the acetylation level of Drp1 to alleviate lipid accumulation and mitochondrial dysfunction induced by Sirt1 on OA.

## Discussion

NAFLD is the most hackneyed chronic liver disease, which is characterized by superabundant fat accumulation [26]. The pathological progression of NAFLD tentatively follows a ‘three-hit’ process, namely, steatosis, lipotoxicity and inflammation [27]. The first step in the development of NAFLD is fat accumulation in the liver, and lipid accumulation can promote lipotoxicity and mitochondrial dysfunction, thus triggering hepatocyte death, inflammation and fibrosis in predisposed patients [28]. Aberrant lipid changes in hepatocytes during hepatic steatosis can directly trigger chronic ER stress in the liver. Higher diacylglyceride, phospholipid, free cholesterol (FC), and free fatty acid (FFA) levels activate ER stress. Lipids can directly induce ER stress through IRE1 and PERK, which sense the biophysical modifications of lipid membranes dependent on the ratio of unsaturated/saturated acyl chains. ER stress activates the mitochondrial apoptosis pathway by destroying Ca^2+^ homeostasis [29, 30]. NAFLD is also linked to chronic inflammation and oxidative stress. Excessive free cholesterol in the livers of diabetic mice with NASH accumulates in mitochondria and the endoplasmic reticulum, leading to an increase in ROS produced by mitochondria and apoptosis in a JNK1-dependent manner [31]. ROS production can also promote hepatic inflammation by increasing the secretion of TNF-α from hepatocytes and KCs, thus upregulating the synthesis of inflammatory cytokines [32].

There is growing evidence that mitochondrial dysfunction is required for the development of NAFLD. Metabolic dysfunction caused by NAFLD leads to mitochondrial dysfunction, which further exacerbates the development of NAFLD. It is well-known that physical exercise (including aerobic exercise and resistance exercise) can reduce liver fat content and effectively alleviate the progression of NAFLD. However, the specific mechanism is unclear and needs to be further explored. In this study, the effect of aerobic exercise on mitochondrial dysfunction induced by NAFLD and its potential mechanism were investigated in rat models of NAFLD. The results show that aerobic exercise can alleviate the liver steatosis and mitochondrial function impairment caused by HFD in rats. The salutary influence of aerobic exercise on mitochondrial dysfunction appears to be associated with activated Sirt1, subsequently decreasing Drp1 acetylation and its activity.

An increasing number of studies have illustrated that exercise can improve the expression of Sirt1 [15, 16]. As a protein deacetylase, Sirt1 is involved in the regulation of multiple cellular pathways [33]. Exercise reduced NAFLD damage caused by HFD by inhibiting lipolysis and enhancing mitochondrial biosynthesis and fatty acid oxidation, and these changes are the result of activation of cellular pathways mediated through Sirt1 [34]. In the constructed zebrafish model of NAFLD, swimming exercise improved hepatic steatosis, inflammation, fibrosis and so on caused by HFD, and these beneficial effects were related to activated Sirt1 signaling [35]. In addition, exercise also alleviates the progression of many diseases by upregulating Sirt1, such as inflammation and metabolic dysfunction of the liver and kidney caused by diabetes [36], myocardial ischemia/reperfusion injury [37] and hypothalamic inflammation [38]. In this study, the level of Sirt1 in rat liver tissue was enhanced by aerobic exercise, and the salutary influence of aerobic exercise on mitochondrial dysfunction caused by HFD was reversed by a Sirt1 inhibitor. At the same time, the same effect of Sirt1 was also shown in the OA cell model. These results are consistent with previous findings that exercise prevents and alleviates NAFLD and its mitochondrial dysfunction by regulating the expression of Sirt1.

Mitochondria are the smallest organelles in cells and the center of energy metabolism and play a momentous role in NAFLD [39]. Mitochondrial biogenesis and fusion/fission are critical in maintaining mitochondrial function [40]. Mitochondrial biogenesis and mitochondrial structural and kinetic alterations have been observed in NAFLD [41]. In NAFLD, the production of superoxide radicals, which are the main source of ROS in mitochondria, is increased. As a result, the production of ROS in mitochondria is increased, which leads to impaired oxidative phosphorylation, reduced ATP and mtDNA production, and ultimately mitochondrial dysfunction [42, 43]. In this study, increased intracellular ROS production, impaired oxidative phosphorylation, reduced ATP and mtDNA production, and decreased mitochondrial membrane potential were observed in a cell model of OA induction. Use of the Sirt1 activator restored the oxidative phosphorylation complex and ATP levels, lessened intracellular ROS generation, and elevated mitochondrial membrane potential and mtDNA copy number. In addition, we examined the protein levels of two factors intimately interrelated to mitochondrial biogenesis, NRF1 and TFAM [44]. The protein levels of NRF1 and TFAM were elevated by the Sirt1 activator. In summary, our results indicated that activation of Sirt1 alleviates mitochondrial dysfunction caused by OA.

Drp1 is a mitochondrial fission-related protein. Abnormal mitochondrial fission mediated by Drp1 leads to ROS overproduction, and inhibition of Drp1 activation can restore mitochondrial function and morphology [45, 46]. Lipid overload has been shown to induce Drp1 acetylation and increase its activity, leading to mitochondrial fission and cardiac dysfunction. Sirt1 is known to be an NAD^+^-dependent deacetylase. PGC-1α activity is promoted through deacetylation, which in turn regulates mitochondrial biogenesis and energy production [47]. Nevertheless, little is known about Sirt1 regulation of Drp1 acetylation and its effects on mitochondrial function. In this research, Drp1 acetylation and its activity were enhanced in an HFD-induced rat model, and increased Drp1 activity was associated with increased levels of its acetylation. Drp1 acetylation and its activity were reduced with the use of Sirt1 activators, accompanied by recovery of mitochondrial dysfunction. The enhancement of Drp1 acetylation reversed the restorative influence of Sirt1 on mitochondrial dysfunction, suggesting that the alleviative influence of Sirt1 is due to the reduction in Drp1 acetylation.

## Study strengths and limitations

This study studied and discussed the mechanism by which aerobic exercise improves NAFLD, it is hoped to provide a richer theoretical basis for the clinical NAFLD research. Based on animal experiments and cell experiments. However, the mechanism of NALFD in this study is limited to mitochondrial dysfunction, and other mechanisms are not involved in in-depth research, such as ER stress and oxidative stress. In addition, the pathogenesis of NAFLD is complex, which can only be truly clarified by multi-organ joint studies, which may be the focus of our future research.

## Conclusion

This study shows that aerobic exercise alleviates hepatic lipid accumulation and improves mitochondrial dysfunction in HFD-fed rats and OA-treated HepG cells. The salutary effect of aerobic exercise is exerted by activating the expression of Sirt1 and restraining the acetylation and activity of Drp1. These results are helpful to clarify the mechanism of aerobic exercise in alleviating NAFLD and its mitochondrial dysfunction and offer a novel target for the ancillary remedy of clinical NAFLD, which reveals the importance of proper aerobic exercise for NAFLD patient care.

## Data Availability

The datasets used and/or analyzed during the current study are available from the corresponding author upon reasonable request.
